# The Cytokine Profiles and Immune Response Are Increased in COVID-19 Patients with Type 2 Diabetes Mellitus

**DOI:** 10.1155/2021/9526701

**Published:** 2021-01-02

**Authors:** Mao Zheng, Xiaobing Wang, Hui Guo, Yinguang Fan, Zichen Song, Zhaohui Lu, Jinquan Wang, Changcheng Zheng, Lin Dong, Yan Ma, Yuyou Zhu, Haoshu Fang, Shandong Ye

**Affiliations:** ^1^The First Affiliated Hospital of USTC, Division of Life Sciences and Medicine, University of Science and Technology of China, Hefei, Anhui 230001, China; ^2^The First Affiliated Hospital of USTC, Division of Life Sciences and Medicine, University of Science and Technology of China, Hefei, Anhui 230031, China; ^3^Department of Breast and Thyroid Surgery, Union Hospital, Tongji Medical College, Huazhong University of Science and Technology, Wuhan, Hubei 430000, China; ^4^Department of Epidemiology and Biostatistics, School of Public Health, Anhui Medical University, Hefei, Anhui 230032, China; ^5^Department of Pathophysiology, Anhui Medical University, Hefei, Anhui 230032, China

## Abstract

The induction of inflammation and cytokine storm was proposed to play a critical role in COVID-19. This study is aimed at investigating the relationship between glucose metabolism and the inflammatory state of inpatients with COVID-19. 71 inpatients with COVID-19 were classified into nondiabetes mellitus (NDM) group, impaired fasting glucose (IFG) group, and diabetes mellitus (DM) group. The average hospitalization days were significantly shorter in DM patients when compared with patients in the IFG group and NDM group. CD4^+^ T cell percentage was higher while CD8^+^ T cells percentage was lower in the DM group than those in the NDM group. The serum levels of IL-6, IL-2, IL-10, and INF-*γ* in the DM group were upregulated when compared with those in the NDM group. The serum levels of TNF-*α*, IL-4, IL-2, IL-10, and INF-*γ* were significantly higher in the DM group than those in the IFG group. A significant difference was observed in CD4^+^ T cell, CD4^+^/CD8^+^ ratio percentage, IL-6, and IL-10 between the NDM group and DM group with adjusted BMI. In conclusion, COVID-19 patients with elevated glucose levels have promoted cytokine profiles and immune response.

## 1. Introduction

The current outbreak of coronavirus disease 2019 (COVID-19), caused by severe acute respiratory syndrome coronavirus 2 (SARS-CoV-2), reached pandemic all over the world in March 2020 [1]. As of June 26, 2020, COVID-19 has been confirmed in 9,413,289 people in 216 countries, carrying a mortality of approximately 5.13% (482,730 cases) [2].

Sepsis was the most frequently observed complication in COVID-19 patients, which might be caused directly by SARS-CoV-2 infection [3]. It has become well accepted that sepsis consists of two, often concomitant phases: a proinflammatory phase with activated immune system and anti-inflammatory phase with immunesuppression [4]. Activated lymphocytes eliminate the invading pathogens through inflammatory cytokines and specific antibodies [5]. Accumulating evidence suggests that a subgroup of patients with severe COVID-19 might have a cytokine storm syndrome, which was proposed to play a critical role in the pathogenesis of COVID-19 [6]. Wang et al. reported that the ICU patients with COVID-19 infection had higher plasma levels of IL2, IL-7, IL-10, and TNF-*α* levels [7]. Especially, significant increased IL-6 levels might be associated with the mortality due to the virally driven hyperinflammation [3]. The investigation of the pathophysiological mechanisms of immunoparalysis in sepsis will be beneficial in optimizing the therapeutic strategy of sepsis.

The overall proportion of diabetes in COVID-19 patients was from 7.4% to 20% [1, 8–10]. Diabetes has been reported as a high risk of death in two earlier coronavirus infections of SARS [11] and the Middle East respiratory syndrome (MERS) [12]. Data about the immune and inflammatory response of COVID-19 in patients with diabetes is limited at present. Increased inflammatory susceptibility and enhanced disease severity is observed in patients of COVID-19 with diabetes, which is associated with increased intensive care unit admission. The cytokine storm is often associated with dysregulation in glucose metabolism, which results in a metabolic and energetic failure [13]. The hyperglycemia is thought to provide glucose to leukocytes without the support of insulin; therefore, the glucose uptake of leukocytes may not be influenced in patients with insulin resistance or insulin deficiency [14]. In the present study, we investigated the incidence of abnormal glucose metabolism in COVID-19 patients and to discuss whether there are any differences in immune and inflammatory response of patients with or without diabetes.

## 2. Research Design and Methods

### 2.1. Study Design and Participants

71 inpatients with COVID-19 from 2020, February 15^th^ to 2020, March 14^th^ in Tumor Center of Wuhan Union Hospital of China were recruited in this retrospective study. Diagnosis and discharge standards of COVID-19 were based on Diagnosis and Treatment of COVID-19 (7^th^ edition) issued by the National Health Commission of the People's Republic of China [15]. All the patients were either SARS-CoV-2 nucleic acid positive, or typical CT imaging features, or SARS-CoV-2 special IgM antibody and IgG antibody positive [15]. Part of the population was patients transferred from clinics or medical shelters because of the worsened illness. The diagnosis criteria of severity type were described in Table A.1.

As described in Table A.2, patients were stratified into three groups of nondiabetes mellitus (NDM), impaired fasting glucose (IFG), and diabetes mellitus (DM), which are based on the fasting blood glucose (FBG), glycosylated hemoglobin A1c (HbA1c), and history of diabetes. IFG or DM was considered according to the diagnostic criteria of Standards of Medical Care in Diabetes-2020 [16]. The history of diabetes was supplied by patients themselves. If elevated FBG or random blood glucose was detected in a patient without a history of abnormal glucose metabolism, the blood glucose was checked once more and further detection of HbA1c was performed in order to determine the classification. Among 71 patients, 6 cases had a preexisting diabetes history and 8 cases were newly diagnosed with diabetes. The design diagram of this retrospective study was shown in Figure S1.

The research was conducted according to the principles of the Declaration of Helsinki and was approved by the Ethics Committee on Clinical Research of The First Affiliated Hospital of USTC. Given the urgency of the COVID-19 pandemic, the informed consent forms were waived by the Ethics Committee.

### 2.2. Data Collection

Demographic information, course of disease duration, medical history, height and weight, medical examinations, and lab reports were obtained from the Electronic Patient Records. All data were checked by two physicians (MZ and XW). The height and weight were measured right at admission. Body mass index (BMI) was calculated by the formula: BMI (kg/m^2^) = weight (kg)/square of height (m). All the test reports were interpreted following the reference value in the manual of agent kits.

### 2.3. Laboratory Procedures

Throat-swab specimens from the upper respiratory tract that were obtained at admission. The novel coronavirus nucleic acid was confirmed by real-time RT-PCR (cobas® z 480 Automatic Fluorescence Quantitative PCR, Roche, USA) with a commercial kit (Pfizer, USA). FBG was determined by the glucose oxidase method (Abbott original reagent, by Architect c16000, Abbott, USA), and HbA1c was tested by high-performance liquid chromatography (Bio-Rad original reagent, by Bio-Rad VARIANT II, Bio-Rad, USA). To identify the immune and inflammatory characteristics, peripheral blood samples were collected for detection of T cell subsets (CD4^+^, CD8^+^) (Beckman Coulter original reagent, by Cytomics FC 500 Flow Cytometer, Beckman Coulter, USA) and cytokines (IL6, TNF*α*, IL2, IL4, IL10, and IFN*γ*), using flow cytometry (commercial kits of BD, USA, by Cytomics FC 500 Flow Cytometer, Beckman Coulter, USA). The peripheral blood of patients was sampled before they used Traditional prescription [17, 18] (“Xinguan No.1”, “Xinguan No.2”, or “Xinguan No.3”), tocilizumab [19] and glucocorticoids [20], considering their effects on inflammation or immune regulation. All indices were tested immediately or on the next morning after admission.

### 2.4. Data and Resource Availability

The datasets analyzed during the current study are available from the corresponding author upon reasonable request. No applicable resources were generated or analyzed during the current study.

### 2.5. Statistical Analyses

Continuous variables are expressed as mean ± SD (normal distribution) or median (Q1, Q3) (skewed distribution). One-way ANOVA test was used for normally distributed data, and Kruskal-Wallis *H* test was used for skewed distributed data to detect differences among the three groups. Count data are presented as percentages. Chi-square test and Fisher's exact test were used to compare the percentages among groups. Natural-log transformations were applied to average serum levels of cytokines to normalize distributions. Multiple linear regression analysis of T cell subsets or cytokines was performed with adjustment for BMI. *P* < 0.05 was regarded as significant. All statistical analyses were performed using SPSS 23.0 (SPSS Inc., Chicago, IL, USA).

## 3. Results

The patients' characteristics were described in Table A.3. The information of therapeutic medicine that patients had been receiving on admission was listed as follows (shown as case (%)): antibiotics drugs 53 (74.65%); antiviral drugs 44 (61.97%); Lianhuaqingwen capsules 33 (46.4%); antihypertensive drugs including Angiotensin Receptor Blocker 3 (4.23%), Calcium Channel Blockers 6 (8.45%), and *β*-blockers 2 (2.82%); diuretic 2 (2.82%); lipid-lowering drugs 1 (1.41%); Sodium bicarbonate 1 (1.41%); Benzbromarone 1 (1.41%); Febuxostat 1 (1.41%); digitalis 1 (1.41%); Sacubitril Valsartan Sodium 1 (1.41%); glucose-lowering medication including metformin 4 (5.63%), acarbose 3 (4.23%), sodium-dependent glucose transporters 2 inhibitors (SGLT2-I) 3 (4.23%), and glimepiride 1 (1.41%).

14 (19.72%) patients with COVID-19 in our wards diagnosed DM and 18 (25.35%) patients were classified into IFG. All the COVID-19 patients with DM were type 2 diabetes mellitus (T2DM).  Table 1 showed the characteristics of COVID-19 patients with different glucose metabolism state. The average age was older in the DM group than it in the NDM group (*P* < 0.05). Two patients in the DM group were diagnosed with a severe type of COVID-19. One patient with DM died of severe heart failure and lactic acidosis after 11 h of admission. Another patient with lung cancer in the NDM group died of severe respiratory failure and refuse of trachea intubation on the 12th day after admission. The average hospitalization days were significantly shorter in patients with T2DM than those in patients with IFG and NDM (*P* < 0.05).  Table 2 showed the glucose-lowering medicine of patients with COVID-19 and T2DM after admission. All the patients in DM groups had no history or evidence of severe diabetic complications.

No differences in various blood cell counts were found among the three groups (Table 1). As shown in Table 3, the percentages of CD4^+^ T cells were higher while CD8^+^ T cell percentage was lower in the DM group than those in the NDM group (*P* < 0.05). Significantly increased CD4^+^ T cell percentage was observed in the DM group when compared to the IFG group (*P* < 0.05). The distribution of T cell subsets was shown in Table A.4. The increased proportion of CD4^+^ T cell percentage was higher, and that of CD8^+^ T cell percentage was lower in the DM group than those in the other two groups without significant difference (*P* > 0.05). The increased proportion of CD4^+^/CD8^+^ T cell ratio was significantly higher in the DM group than that in the NDM group (*P* < 0.05). After adjustment for BMI, CD4^+^ T cell percentage was lower in the NDM and IFG groups than that of the DM groups, meanwhile CD4^+^/CD8^+^ ratio was lower in the NDM group than that in the DM group (*P* < 0.05).

**Figure 1:**
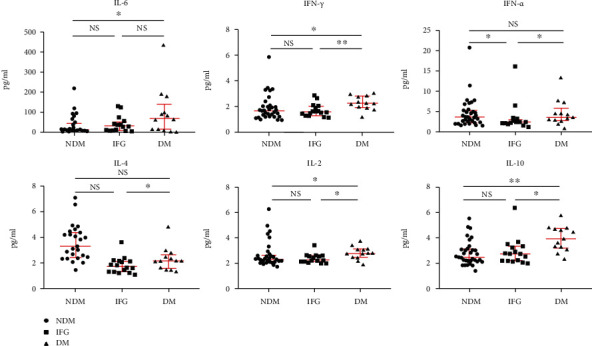
The cytokines levels among the NDM, IFG, and DM groups. NDM: nondiabetes mellitus; IFG: impaired fasting glucose; DM: diabetes mellitus. ^∗^*P* < 0.05 and ^∗∗^*P* < 0.005. Black circle = NDM group (*n* = 33); black square = IFG group (*n* = 16); black triangle = DM group (*n* = 13). Because the cytokines levels of 9 patients were not performed, data of 62 patients were included.

In Figure S2, we showed the population distribution of different levels of cytokines. Most patients had elevated IL-6 levels. As shown in Figure 1, the serum levels of IL-6, IL-2, IL-10, and INF-*γ* in the DM group were upregulated when compared with those in the NDM group (*P* < 0.05); the serum levels of TNF-*α*, IL-4, IL-2, IL-10, and INF-*γ* were significantly lower in the IFG group than those in the DM groups (*P* < 0.05); TNF-*α* level was lower in the NDM group than that in the IFG group (*P* < 0.05). Considering the correlation between obesity and inflammation, we tried to analyze the data with an adjustment for BMI. The result showed that the levels of IL-6 and IL-10 were lower in the NDM group, as well as INF-*γ* and IL-10 were significantly lower in the IFG group than those in group DM (*P* < 0.05) with adjustment for BMI (Table 4).

## 4. Discussions

DM was reported as one of the most common coexisting conditions in COVID-19. The incidence of DM among patients with COVID-19 ranged from 7.4% to 20% [1, 8–10]. A recent meta-analysis retrieved 12 studies of 2108 Chinese patients with confirmed SARS-Cov-2 infection and showed that the prevalence of diabetes was 10.3% [21]. In our data, 19.72% of patients were diagnosed with T2DM, and 25.35% of patients were classified into IFG. The incidence was higher than in some other studies [8–10], which can be explained by a combination of blood glucose and HbA1c used as the diagnostic criteria of diabetes [16]. One major epidemiological investigation showed that the prevalence of diabetes and prediabetes in Chinese adults was estimated to be 10.9% and 35.7%, respectively, in 2013 [22]. It is still unclear whether the patients with diabetes are more likely to get COVID-19, but at least, the incidence of abnormal glucose metabolism in COVID patients in our ward seemed consistent with the general population. A recent meta-analysis recruited the studies from China, and Italy concluded that diabetes may not increase the risk of SARS-CoV-2 infection [21]. However, with the rapidly evolving situation, close monitoring of these data is important to the final conclusion. The prevalence of diabetes in SARS and MERS was also referred. Chen et al. reported that 23.89% (16 cases) of 67 patients with SARS had diabetes [23]. Fan et al. reported that 34.30% (83 cases) of 242 patients with SARS had a diabetes history or FBG ≥ 7.0 mmol/L [24]. In a meta-analysis of MERS, diabetes was prevalent in 51 ± 8% (from 10%~77%) of patients [25].

Most of the reports we referred to showed patients with Diabetes in SARS and MERS had higher mortality than those without abnormal glucose metabolism [11, 12, 23, 24]. Diabetes was considered to be associated with adverse outcomes, greater ICU admissions, higher rates of ventilation, and increased mortality in patients of COVID-19 [26]. One dissenting voice was a study by Zhang et al., considering diabetes was not a risk for severe disease in COVID-19 [27]. In a recent study by Zhu et al., preexisting T2DM patients with better-controlled blood glucose had significantly reduced risk of all-cause mortality and detrimental complications than those with poorly controlled blood glucose [28]. Zumla et al. proposed a concept that host-directed therapy can be applied to the COVID-19 treatment [29]. Several marketed antidiabetic drugs with excellent safety profiles such as metformin and glitazones, as well as insulin, could reduce immunopathology and prevent or curb inflammation response [29, 30]. As early as the year 2005, a large, landmark study by the team of Greet Van den Berghe provided clinical evidence that the anti-inflammatory effects of insulin probably play an important role in the improvement of clinical adverse outcomes in acute illness [31, 32]. The anti-inflammatory properties of metformin were also proved to exert irrespective of diabetes mellitus status [33]. A recent review summarizes available data from present observational retrospective studies that have shown a reduction in mortality in metformin users compared with nonusers and analyzed the pleiotropic effects of metformin such as the improvement of glucose, reduction in body weight and insulin resistance, and also inhibition of multiple pathogenic mechanisms that contribute to worsen the prognosis of patients with COVID-19 [34]. In our study, most patients with diabetes were moderate type of COVID-19, and majority of them had favorable prognosis. The average hospitalization duration of patients with diabetes was significantly shorter than those without diabetes. This phenomenon may be explained partly by the good control of blood glucose and no severe diabetic complications in our study. Half of our patients with diabetes got the HbA1c lower than 7.5% (58 mmol/mol). After adjustment of glucose-lowering regiment, most patients reached a desired glucose level of FBG between 4-8 mmol/L and postprandial blood glucose between 6-12 mmol/L. All the preexisting T2DM patients and newly-diagnosed patients had no history or evidence of severe diabetic complications. Also, most of the patients with COVID-19 and T2DM received metformin and insulin therapy as a glucose-lowering regiment during their hospitalization. The benefits of anti-inflammation and immune regulation of these two medicines could be the second reason for the shortened hospitalization days and favorable prognosis.

T helper cells (CD4^+^) are simulated by antigens and differentiate into different subtypes of T cells to participate in cell-mediated and/or humoral immunity, while CD8^+^ cytotoxic T lymphocytes play a pivotal role in recognition of endogenous antigen peptides and elimination of infected cells [35]. Our study showed the immunological changes in COVID-19 patients with DM, who presented a higher percentage of CD4^+^, but a lower percentage of CD8^+^ than those in nondiabetic patients. Most of the patients [52 (73.24%)] presented nonincreased CD4^+^ percentage, and 19 (26.76%) of patients presented increased abnormal CD4^+^ percentage, especially in the patients with DM (Table A.4), which was not completely identical to the results of other studies [36, 37]. Qin et al. [36] reported that the decrease of CD4^+^ T cells and no significant change in the number of CD8^+^ cells were common among patients with COVID-19, especially in severe cases. Chen et al. [37] reported that lower CD4^+^ T cell count was an indicator of immunosuppression and was significantly associated with ICU admission. Therefore, COVID-19 might damage T lymphocytes, and the immune disorders occurred during the period of disease, especially in patients with DM. But how the T helper cells and suppressor T cells act in the pathogenesis of COVID-19 needs more researches to elucidate.

Activated CD4^+^ T cells secrete either inflammatory cytokines (TNF-*α*, INF-*γ*, IL-6, and IL-2) or cytokines with anti-inflammatory cytokines (Th2: IL-4, IL-10) [35]. The imbalance of the immune system leads to inflammation, which is a response of an organism to virus infection. In the lungs from severe COVID-19 patients, a large amount of inflammatory cell infiltration was observed in pulmonary pathology [38]. The study by Zhou et al. showed that after the SARS-CoV-2 infection, inflammatory CD14^+^CD16^+^ monocytes highly expressed IL-6 and accelerated the inflammation [19]. The concept of excessive inflammatory reaction and cytokine storm was proposed to play a pivotal role in the pathogen mechanism of COVID-19, which may be similar to the pathogenesis of SARS or MERS [6]. IFN-*γ*, TNF-*α*, and IL-6 have been confirmed to be critical pathogenic cytokines involving in the inflammatory storm in patients infected with SARS-CoV [39] or MERS-CoV [40].

Low-graded chronic inflammation is now recognized as an important feature of T2DM. Cytokines (including IL-6, IL-8, and TNF-*α*) act as a proinflammatory cytokine and a coinducer in the development of insulin resistant and *β*-cell dysfunction, which precedes the development of T2DM [41, 42]. The concept that targeting inflammation to improve insulin sensitivity, *β*-cell function, and glucose metabolism was widely accepted [42]. IL-6 regulated the expression of anti-inflammatory like IL-10 and INF-*γ* and also inhibited the expression of TNF-*α* and IL-1*β* [41, 43]. In this study, the serum levels of IL-2, IL-10, and INF-*γ* were higher in COVID-2019 patients with diabetes than both patients with IFG and patients with normal sugar. The IL-6 level of the DM group and the TNF-*α* level of the IFG group were upregulated than these two indicts in the NDM group.

As obesity is associated with chronic inflammation [44], we adjusted BMI for T cell subsets and cytokines. CD4^+^ T cells and CD4^+^/CD8^+^ ratio percentages, as well as IL-6 and IL-10, showed higher levels in the DM group than those in the NDM group; meanwhile, the CD4^+^ T cell percentage and IL-10 were higher in the DM group than those in the IFG group, which suggested the increased inflammatory response presented in COVID-19 patients with hyperglycemia. In view of the pleiotropic effects of IL-6, we proposed that when the body was infected by the coronavirus, the increased blood glucose might be beneficial for anti-inflammatory cytokines (like IL-10 and IFN-*γ*) and elimination of the virus, which could explain short average hospitalization days of DM patients in this study. Adaptive elevated levels of IL-6 represent a compensatory mechanism in order to reduce inflammation and maintain proper glucose homeostasis [41].

There were several limitations in our study which might make potential bias. Firstly, it was a retrospective, single-center study with limited patient number. The collection of standardized data for a larger cohort would help to further draw clear, even robust conclusions. So, the main value of our conclusion perhaps is simply a contribution of data specific to diabetes patients observed in China. Secondly, postprandial blood glucose and HbA1c were not tested in each patient, which may lead to the missed diagnosis of impaired glucose regulation; Third, even though DM patients had higher cytokines, the prognosis was not worse than patients of COVID-19 without diabetes. So, it is hard to use the inflammatory and immune index in our study as an important biomarker to identify the severity of COVID-19.

In conclusion, COVID-19 patients with elevated glucose levels have promoted cytokine profiles and immune responses. The favorable prognosis of patients with moderate COVID-19 and T2DM, especially for those without severe diabetic complications in our study, may partly be due to the use of glucose-lowering medicine such as metformin and/or insulin. However, firm conclusions can only be drawn from double-blind randomized controlled trials (RCTs), and such RCTs are very difficult to perform in patients with COVID-19.

## Figures and Tables

**Table 1:** Comparison of characteristics, immune response, and cytokines of patients in different groups.

	NDM	IFG	DM
*N* (%)	39 (54.93)	18 (25.35)	14 (19.72)
Age (y)	54.31 ± 14.35	61.28 ± 14.00	63.00 ± 10.03^∗^
Female (%)	24 (61.54)	10 (55.56)	5 (35.71)
BMI (kg/m^2^)	22.11 ± 3.15	22.86 ± 2.76	24.34 ± 2.57^∗^
Hospitalization duration (d, 2 deaths were removed)	22.00 (19.00, 27.00)	21.00 (17.50, 23.00)	13.00 (9.00, 17.50)^∗^^/†^
Duration from illness onset to discharge (d, 2 deaths were removed)	35.97 ± 9.47	26.67 ± 8.11^∗^	27.00 ± 11.16^†^
Severity of illness			
Moderate *N* (%)	38 (97.44)	18 (100)	12 (85.71)
Severe *N* (%)	1 (2.56)	0 (0)	2 (14.29)
Death *N* (%)	1 (2.56)	0 (0)	1 (7.14)
Laboratory finding			
White blood cell (G/L)^‡^	4.97 (4.34, 6.05)	5.13 (4.42, 6.11)	5.91 (4.54, 7.62)
Neutrophil (G/L)^‡^	2.98 (2.49, 4.08)	3.25 (2.58, 4.49)	3.42 (2.98, 5.09)
Lymphocyte (G/L)	1.43 ± 0.46	1.25 ± 0.37	1.36 ± 0.54
Monocyte (G/L)^‡^	0.44 (0.34, 0.60)	0.49 (0.39, 0.63)	0.47 (0.32, 0.52)
Lymphopenia *N* (%)	11 (28.21)	8 (44.44)	7 (35.71)
IL-6/IL-10	3.94 (1.92, 14.82)	12.03 (3.16, 18.05)	17.61 (3.51, 33.20)
SARS-CoV-2 nucleic acid positive *N* (%)	16 (41.03)	8 (44.44)	8 (57.14)
SARS-CoV-2 IgM antibody (AU/ml)^‡^	35.08 (10.37, 77.25)	36.99 (12.55, 66.51)	62.75 (36.76, 75.87)
SARS-CoV-2 IgG antibody (AU/ml)^‡^	156.83 (133.95, 169.74)	160.16 (133.70, 180.71)	176.06 (125.81, 187.96)

NDM: nondiabetes mellitus; IFG: impaired fasting glucose; DM: diabetes mellitus; ^∗^ vs. NDM group, *P* < 0.05; ^†^vs. IFG group, *P* < 0.05. ^‡^Kruskal Wallis Test was performed when the data presented skewed distribution. One patient with DM died of severe heart failure and lactic acidosis after 11 h of admission. Another patient with lung cancer in the NDM group died of severe respiratory failure and refuse of trachea intubation on the 12th day after admission.

**Table 2:** Glucose-lowering medication in patients of COVID-19 with T2DM.

Glucose-lowering medication	Case (%)
Metformin	12 (16.90%)
Insulin	7 (9.86%)
Acarbose	6 (8.45%)
SGLT2-I^∗^	2 (2.82%)
Pioglitazone	1 (1.41%)

^∗^SGLT2: sodium-dependent glucose transporters 2 inhibitors.

**Table 3:** T cell subsets among NDM, IFG, and DM.

Groups	NDM	IFG	DM
CD4^+^ T cell percentage (25.34-51.37%)	43.63 ± 9.97	43.81 ± 9.05	51.75 ± 4.45^∗^^/†^
CD8^+^ T cell percentage (14.23-38.95%)	26.50 ± 8.18	24.49 ± 10.02	20.95 ± 7.61^∗^
CD4^+^/CD8^+^ (0.41-2.72)^‡^	1.64 (1.28, 2.41)	1.86 (1.30, 2.91)	2.80 (1.79, 3.71)

NDM: nondiabetes mellitus; IFG: impaired fasting glucose; DM: diabetes mellitus; ^∗^ vs. NDM group, *P* < 0.05; ^†^vs. IFG group, *P* < 0.05. ^‡^Kruskal Wallis Test was performed. NDM group (*n* = 33); IFG group (*n* = 16); DM group (*n* = 13). Because the T cell subsets of 2 patients were not performed, data of 69 patients were included.

**Table 4:** Multiple linear regression analysis of T cell subsets and cytokines adjusted with BMI.

	Group	*β*	SE	Adjust *β*	*t*	*P*
LgIL-6	DM	0 (ref.)				
	NG	-0.45	0.19	-0.39	-2.37	0.02
	IGR	-0.28	0.21	-0.22	-1.35	0.18
LgINF*γ*	DM	0 (ref.)				
	NG	-0.09	0.06	-0.27	-1.64	0.11
	IGR	-0.12	0.06	-0.34	-2.10	0.04
LgTNF*α*	DM	0 (ref.)				
	NG	-0.00	0.09	-0.00	-0.01	0.99
	IGR	-0.14	0.10	-0.23	-1.37	0.18
LgIL-4	DM	0 (ref.)				
	NG	-0.03	0.06	-0.08	-0.49	0.63
	IGR	-0.09	0.07	-0.22	-1.32	0.19
LgIL-2	DM	0 (ref.)				
	NG	-0.03	0.04	-0.12	-0.68	0.50
	IGR	-0.07	0.04	-0.28	-1.71	0.09
LgIL-10	DM	0 (ref.)				
	NG	-0.14	0.04	-0.48	-3.10	0.00
	IGR	-0.13	0.05	-0.40	-2.68	0.01
CD4	DM	0 (ref.)				
	NG	-7.31	3.03	-0.41	-2.42	0.02
	IGR	-7.28	3.32	-0.36	-2.19	0.03
CD8	DM	0 (ref.)				
	NG	5.29	3.02	0.30	1.75	0.09
	IGR	3.76	3.31	0.19	1.14	0.26
LgCD4/CD8	DM	0 (ref.)				
	NG	-0.18	0.07	-0.41	-2.45	0.02
	IGR	-0.13	0.08	-0.27	-1.67	0.10

NDM: nondiabetes mellitus; IFG: impaired fasting glucose; DM: diabetes mellitus.

## Data Availability

The data used to support the findings of this study are available from the corresponding author upon request.

## Additional Points

*Highlight*. (i) In the present study, we observed promoted cytokine profiles and immune response in moderate COVID-19 patients with elevated glucose levels on admission. (ii) Of note, most patients with COVID-19 and T2DM had desirable prognosis in our study, indicated by significantly shortened hospitalization time. (iii) The favorable prognosis may partly be due to the use of glucose-lowering medicine as metformin and/or insulin. *Guarantor Statement*. Professor Fang H and professor Ye S are the guarantors of this work and, as such, had full access to all the data in the study and takes responsibility for the integrity of the data and the accuracy of the data analysis.

## Supplementary Materials

Supplementary Materials Table A.1: the diagnosis criteria of severity types. Table A.2: the classification of patients with COVID-19 according to blood glucose and HbA1c. Table A.3: the characteristics of patients with COVID-19. Table A.4: distribution of T cell subset percentages among the NDM, IFG, and DM groups. Figure A.1: design diagram of the study. Figure A.2: population distribution of cytokines in patients with COVID-19. Green sector = normal group; blue sector = missed data; red sector = elevated level. The cutoff of each index was set according to the reference value in the manual of the reagent kit.
