# Supplementary material for: The Cytokine Profiles and Immune Response Are Increased in COVID-19 Patients with Type 2 Diabetes Mellitus
Source: J Diabetes Res. 2021 Jan 2;2021:9526701. doi: 10.1155/2021/9526701 (PMC7796849; doi:10.1155/2021/9526701)
Supplement: Supplementary Materials — Table A.1: the diagnosis criteria of severity types. Table A.2: the classification of patients with COVID-19 according to blood glucose and HbA1c. Table A.3: the characteristics of patients with COVID-19. Table A.4: distribution of T cell subset percentages among the NDM, IFG, and DM groups. Figure A.1: design diagram of the study. Figure A.2: population distribution of cytokines in patients with COVID-19. Green sector = normal group; blue sector = missed data; red sector = elevated level. The cutoff of each index was set according to the reference value in the manual of the reagent kit. [file 9526701.f1.doc]

**Table A.1 The diagnosis criteria of severity types**

| **Type** | **Criteria** |
| --- | --- |
| Mild | The clinical symptoms are mild, and no signs of pneumonia are found on the imaging |
| Moderate | Patients have fever and respiratory symptoms, and signs of pneumonia can are found on the imaging |
| Severe | Shortness of breath, RR≥30 breaths/min  or oxygen saturation≥93% at rest  or arterial oxygen partial pressure (PaO2)/oxygen absorption concentration (FiO_2_)≤300mmHg(1mmHg=0.133kPa)  or imaging shows a progression of pulmonary lesions more than 50% within 24 to 48 hours |

**Table A.2 The classification of patients with COVID-19 according to blood glucose and HbA1c**

|  | **Groups** | | |
| --- | --- | --- | --- |
|  | **NDM** | **IFG** | **DM** |
| **Diagnosis criteria** | FBG＜ 5.6mmol/L | FBG 5.6-6.9mmol/L | 1. FBG≥7mmol/L, Or random blood glucose≥11.1mmol/L   And 2. HbA1c ≥ 6.5% (48 mmol/mol) |

NDM: non-diabetes mellitus; IFG: impaired fasting glucose; DM: diabetes mellitus; FBG: fasting blood glucose; HbA1c: glycosylated hemoglobin A1c

**Table A.3 The characteristics of patients with COVID-19**

| **Characteristics** | **Total** |
| --- | --- |
| **N** | 71 |
| **Age (y)** | 57.79±13.91 |
| Young people (≤44y) N (%) | 11 (15.49) |
| Middle-aged people (45-59 y) N (%) | 25 (35.21) |
| Older people (≥60y) N (%) | 35 (49.30) |
| **Sex** |  |
| Female (%) | 39 (54.93) |
| BMI (kg/m^2^) | 22.74±3.03 |
| **Main Signs and symptoms at admission** |  |
| Fever N (%) | 50 (70.42) |
| Dry cough N (%) | 19 (26.76) |
| Chest stuffiness (%) | 10 (14.08) |
| Palpitation N (%) | 8 (11.27) |
| Diarrhea N (%) | 4 (5.63) |
| **Comorbidities** |  |
| High blood pressure (%) | 7 (9.86) |
| Coronary heart disease (%) | 2 (2.82) |
| Dilated cardiomyopathy (%) | 1 (1.41) |
| Chronic heart failure (%) | 2 (2.82) |
| Chronic renal failure (%) | 1 (1.41) |
| Lung cancer (%) | 1 (1.41) |
| **Duration from illness onset to admission or transfer (d)** | 10.0 (7.0, 15.0) |
| **Hospitalization duration (d, 2 deaths were removed) ^a^** | 20.0 (16.5, 24.0) |
| **Duration from illness onset to discharge^*^**  **(d, 2 deaths were removed)** | 32.12±10.29 |
| **Severity of illness** |  |
| Moderate N(%) | 68 (95.77) |
| Severe N(%) | 3 (4.23) |
| Death N(%) | 2 (2.82) |
| **Laboratory finding** |  |
| White blood cell (G/L) | 5.14(4.42, 6.50) |
| Neutrophil (G/L) | 3.21(2.59, 4.10) |
| Lymphocyte (G/L) | 1.37±0.46 |
| Monocyte (G/L) | 0.47(0.37, 0.59) |
| Lymphopenia N (%) | 24 (33.80) |
| CD4^+^ (%) | 45.21±9.39 |
| CD8^+^ (%) | 24.93±8.73 |
| CD4^+^/CD8^+^ | 1.82 (1.38, 2.80) |
| SARS-CoV-2 nucleic acid positive (%) | 32 (45.07) |
| SARS-CoV-2 IgM antibody (AU/ml) | 40.96(13.63,74.35) |
| IgM positive (%) | 82.54 |
| SARS-CoV-2 IgG antibody (AU/ml) | 159.92(131.51, 79.83) |
| IgG positive (%) | 93.65 |
| IL-6 (pg/ml） | 16.70 (6.29, 68.85) |
| TNF-α (pg/ml） | 3.06 (2.29, 4.83) |
| sIL-4 (pg/ml） | 1.89 (1.45, 2.28) |
| IL-2 (pg/ml） | 2.42 (2.12, 2.75) |
| IL-10 (pg/ml） | 2.78 (2.22, 3.65) |
| INF-γ (pg/ml） | 1.75 (1.33, 2.26) |

^*^ The date of meeting discharge criteria was used as the ending of the study;

^a^ Discharge criteria (17): body temperature returns to normal for more than 3 days; respiratory symptoms improved significantly; pulmonary imaging showed significant improvement in acute exudative lesions;Two consecutive throat swabs showed SARS-CoV-2 nucleic acid positive (sampling time interval at least 24 hours).

**Table A.4 Distribution of T cell subsets percentages among NDM, IFG and DM groups.**

|  | | Non-increased proportion  Patients’ number (%) | Increased proportion  Patients’ number (%) | *P* value | *P* value of trend test |
| --- | --- | --- | --- | --- | --- |
| CD4^+^ T cell | Total | 50 (72.46) | 19 (27.54) | 0.285^*^ | 0.23 |
|  | NDM | 29 (76.32) | 9 (23.68) |  |  |
|  | IFG | 14 (77.78) | 4 (22.22) |  |  |
|  | DM | 7 (53.85) | 6 (46.15) |  |  |
| CD8^+^ T cell | Total | 63 (91.30) | 6 (8.70) | 0.617^*^ | 0.43 |
|  | NDM | 34 (89.47) | 4 (10.53) |  |  |
|  | IFG | 16 (88.89) | 2 (11.11) |  |  |
|  | DM | 13 (100) | 0 (0) |  |  |
| CD4^+^/CD8^+^ratio | Total | 50 (72.46) | 19 (27.54) | 0.067^*^ | 0.02 |
|  | NDM | 31 (81.58) | 7 (18.42) |  |  |
|  | IFG | 13 (72.22) | 5 (27.78) |  |  |
|  | DM | 6 (46.5) | 7 (53.85) |  |  |

NDM: non-diabetes mellitus; IFG: impaired fasting glucose; DM: diabetes mellitus;

The proportions of increased percentage of T cell subsets were defined according to the kit instructions supplied by the manufacturers.

^*^ Fisher’s exact test


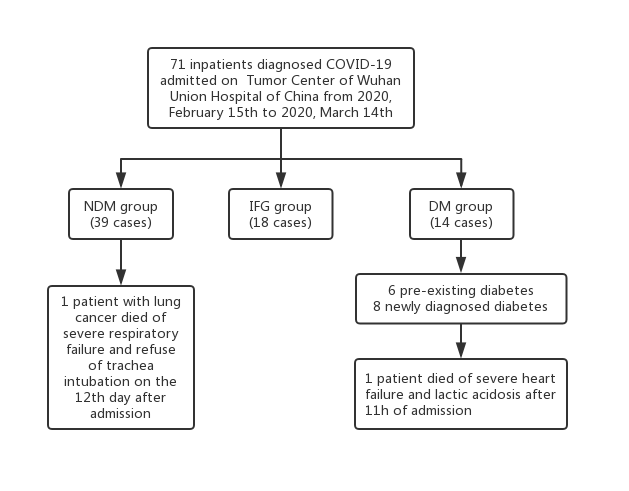


**Figure A.1 Design diagram of the study**


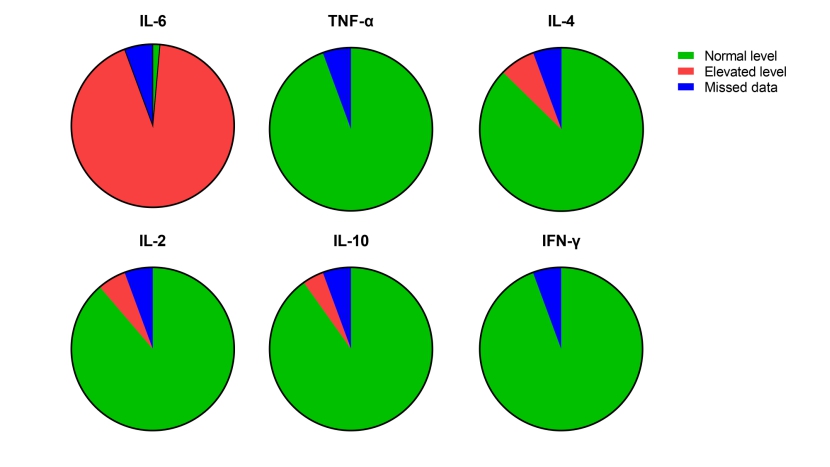


**Figure A.2 Population distribution of cytokines in patients with COVID-19**

Green sector=normal group; blue sector=missed data; red sector=elevated level. The cutoff of each index was set according to the reference value in the manual of reagent kit.
